# SPECT findings on neuropsychiatric symptoms caused by nitrous oxide abuse

**DOI:** 10.3389/fpsyt.2022.980516

**Published:** 2022-11-17

**Authors:** Li Wang, Lijie Yin, Qian Wang, Renbin Wang, Zunjing Liu, Mingrui Dong, Xiaohui Duan, Yumin Zheng, Wen Hong, Fang Liu, Changle Tie

**Affiliations:** ^1^Department of Neurology, The First Hospital of Tsinghua University, Beijing, China; ^2^Department of Neurology, China-Japan Friendship Hospital, Beijing, China; ^3^Department of Nuclear Medicine, China-Japan Friendship Hospital, Beijing, China; ^4^Department of Radiology, China-Japan Friendship Hospital, Beijing, China

**Keywords:** neuropsychiatric symptoms, neuropsychological, regional cerebral blood flow, nitrous oxide abuse, SPECT

## Abstract

**Objective:**

The aim of the study was to investigate the clinical, neuropsychological, and regional cerebral blood flow (rCBF) perfusion changes in patients with neuropsychiatric symptoms caused by nitrous oxide (N_2_O) abuse.

**Methods:**

A total of 16 patients with neuropsychiatric symptoms caused by nitrous oxide abuse were recruited for this study. The study was carried out in the withdrawal phase of N_2_O abuse. A 925–1110 MBq ^99m^Tc-ECD was administered intravenously. SPECT/CT images were collected with a low-energy and high-resolution collimator. The region uptake statistics of different brain regions of interest between patients with N_2_O abuse and normal people of the databases for younger subjects from the Scenium DB Comparison software were calculated automatically.

**Results:**

The clinical manifestations of the 16 patients with neuropsychiatric symptoms were mood lability, anxiety, hallucination, delusion, agitation, confusion, and other psychiatric symptoms. In addition, 15 of the patients also complained of memory decline; 14 patients manifested numbness or paresthesia; 14 patients developed limb weakness, and their motor impairments were more severe in the lower limbs than in the upper limbs; and eight patients had urinary and defecation disturbances. In the neuropsychological examination, the BPRS score was 54.69 ± 11.48, the HAMD score was 30.00 ± 11.06, the HAMA score was 18.06 ± 5.77, the MMSE score was 28.06 ± 2.29, and the MoCA score was 25.06 ± 3.40. SPECT showed hypoperfusion in the frontal and temporal lobes, which is consistent with the clinical findings.

**Conclusion:**

This was the first study to demonstrate the obvious effect of N_2_O abuse on CBF in patients with neuropsychiatric symptoms. CBF perfusion imaging is helpful to detect the changes in the local brain functional activity in patients with N_2_O abuse.

## Introduction

The Global Drug Survey 2019, which was conducted in more than 30 countries, revealed that nitrous oxide (N_2_O) was the 10th most popular substance among the studied population (1). N_2_O abuse for recreation is increasingly popular among Chinese adolescents and young adults, which leads to neurological and psychiatric complications (2). Subacute combined degeneration and peripheral neuropathy are identified as the most common neurological manifestations (3, 4). However, psychiatric and cognitive impairments caused by N_2_O abuse have not received much attention, and only case reports have been reported in the literature (5, 6).

Numerous studies have demonstrated significant mental health and behavioral comorbidities among patients who are inhalant abusers. Inhalant abusers are more likely to have an episode of major depression (7) and suicidality (8) and are at an increased risk of drug abuse problems in future (9). Functional imaging has proven to be very sensitive in detecting cerebral blood flow and metabolism derangement early in the course of drug addiction (10). This work focuses on single-photon emission computed tomography (SPECT) findings in patients with neuropsychiatric symptoms caused by N_2_O abuse.

## Methods

### Subjects

A total of 16 patients who exhibited neuropsychiatric symptoms caused by N_2_O abuse between February 2018 and August 2020 were enrolled. The enrollment criteria for this study are as follows: (1) Patients with a history of N_2_O inhaling; (2) patients whose conditions comply with the diagnostic criteria of inhalant-related disorders, as coded according to the Diagnostic and Statistical Manual of Mental Disorders (DSM-V); and (3) patients with a Brief Psychiatric Rating Scale (BPRS) score >35 points. The exclusion criteria of the study are as follows: patients with neuropsychiatric symptoms caused by other diseases or mental disorders.

This study was approved by the Ethics Committee of the China–Japan Friendship Hospital (trial number 2018-34-K25). Written informed consent was obtained from all the participants. This study was carried out in the withdrawal phase of N_2_O abuse.

### Clinical data collection

All the patients underwent a standard neurologic examination conducted by a neurologist, who registered clinical data and performed neuroimaging studies and laboratory tests. The mental state examination and neuropsychological rating were assessed by a psychiatrist using the following measures: (1) Brief Psychiatric Rating Scale (BPRS), (2) Hamilton Depression Scale (HAMD), (3) Hamilton Anxiety Scale (HAMA), (4) Mini-Mental State Examination (MMSE), and (5) Montreal Cognitive Assessment (MoCA).

### Single-photon emission computerized tomography (SPECT)

SPECT were performed using a conventional dual-head gamma camera system (Symbia T2, Siemens Medical Solutions, Germany) equipped with low-energy high-resolution parallel-hole collimators nearly at the same time as subjective assessment. All the patients were supplemented with methylcobalamin in hospitalization. Brain perfusion imaging was performed for 20 min, followed by an intravenous injection of a 925–1110 MBq ^99m^Tc ethyl cysteinate dimer (ECD) (radiochemical purity >95%, HTA Co. Ltd, Beijing, China) in a dimly lit and quiet room with the patients' eyes closed. Projection images were acquired through a 20% window centered on the 140 keV peak. The scan time per view was 25 s, and the matrix was 256 × 256. Scenium DB Comparison software (from the Siemens symbia.net Neurology software package) was used for neurological evaluations with SPECT/CT imaging, enabling the comparison of functional studies (SPECT) of a specific patient to a database composed of scans from confirmed normal individuals. Each SPECT image was anatomically standardized using the MNI template provided by SPM. Then, the count of each voxel was standardized to the mean voxel count of the whole brain using proportional scaling. Quantitative parametric analysis was performed by database comparison software like Scenium (we choose the databases for younger subjects to analyze SPECT/CT imaging of patients with N_2_O abuse), which provides powerful quantification tools for the assessment of SPECT/CT, performs a voxel-by-voxel evaluation of the abnormal regions, and automatically identifies anatomical regions of interest (ROIs). The neurology workflow provides voxel-based statistics displayed as an image volume and calculates ROI statistics by comparing the corresponding estimated normal population mean with the value observed in the patient. Each SPECT image was segmented into 18 regions of interest (ROI) automatically and calculated by Scenium DB Comparison software. The statistical difference in the average uptake value of each brain region was calculated automatically.

The statistic was calculated by means of equation:


$$\begin{array}{l} statistic=\frac{\left(Patient\;Value\;Population\;Mean\right)}{Population\;Standard\;Deviation} \end{array}$$

A statistic >1.68 indicated an increase in local CBF perfusion, while that < -1.68 indicated a decrease in local CBF perfusion. A statistic was normal between −1.68 and 1.68.

### Statistical analysis

All continuous variables were presented as mean ± standard deviation. After SPECT image reconstruction, database comparison software was used for automatic processing and analysis. In total, 18 brain regions (e.g., basal ganglia, central region, cerebellum, cingulate gyrus, frontal lobe, medial temporal lobe, occipital lobe, parietal lobe, and temporal lobe) were calculated automatically. The paired sample *t*-test was performed on left and right brain regions. One-way ANOVA, followed by the least significant difference (LSD) test, was used to compare the averages and variances among different brain regions. The χ^2^ test was used to compare the changes in regional cerebral blood flow (rCBF) in different brain regions, and a *P*-value < 0.05 was considered statistically significant.

## Results

### Demographic and clinical features

In this study, there were eight male and eight female patients with an age of onset ranging between 18 and 29 (mean age 21.56 ± 2.83) years old. The duration of N_2_O exposure varied from 3 to 36 (mean 17.88 ± 11.23) months. The course of disease varied from 0.5 to 4 (mean 1.81 ± 1.09) months. Nonmedical abuse of more than one substance was found in four patients. Abuse of both N_2_O and cannabinoids was found in cases 1, 7, 8, and 10. A total of eight patients self-medicated with methylcobalamin prior to their hospital admission.

Clinical features of N_2_O abuse resulting in neuropsychiatric disorders are provided in Table 1, including mood lability, anxiety, hallucination, delusion, agitation, confusion, and other psychiatric symptoms. In addition, 15 of the patients complained of memory decline; 14 patients manifested numbness or paresthesia; 14 patients developed limb weakness, with motor impairments found more severe in lower limbs than in upper limbs; and eight patients had urinary and defecation disturbances.

**Table 1:** Clinical features of N_2_O abuse resulting in neuropsychiatric disorders.

**Clinical features**	**Number of patients**	**%**
Confusion	7	43.75
Hallucination	13	81.25
Delusion	11	68.75
Panic attacks	5	31.25
Bizarre behavior	6	37.50
Manic	1	6.25
Agitated	7	43.75
Anxiety	12	75.00
Mood lability	16	100.00
Suicide ideation	4	25.00
Forgetfulness	15	93.75
Numbness or paresthesias	14	87.50
Weakness	14	87.50
Urinary disorder	8	50.00

Laboratory findings are given in Table 2. Vitamin B12 levels were normal (893.00 ± 414.19 pmol/L) in the nine self-medicated patients with methylcobalamin before admission, and homocysteine levels were still high in four of these patients. Among the remaining nine patients who were unmedicated with methylcobalamin before admission, vitamin B12 levels (133.30 ± 64.12 pmol/L) were low in four patients and low to normal in the remaining three patients. Homocysteine levels were high in all the unmedicated patients (62.07 ± 38.73 μmol/L). Anemia was diagnosed in nine patients.

**Table 2:** Laboratory findings of N_2_O abuse resulting in neuropsychiatric disorders.

	**Unmedicated with methylcobalamine before admission (*****N*** = **7)**		**Self-medicated with methylcobalamine before admission (*****N*** = **9)**	
	**Mean ± SEM**	**Range**	**Mean ± SEM**	**Range**
Vitamin B12 (133–675 pmol/L)	133.30 ± 64.12	76.00–237.00	893.00 ± 414.19	221.00–1476
Homocysteine ( ≤ 15 μmol/L)	62.07 ± 38.73	24.68–128.56	18.50 ± 13.06	8.00–47.57
Hemoglobin (115–150 g/L)	116.29 ± 22.69	87.00–158.00	131.56 ± 22.42	103.00–160.00
MCV (82–100 fL)	92.20 ± 6.33	81.70–101.80	93.49 ± 10.68	77.30–112.7

### Neuropsychological rating scale

Data from the neuropsychological rating scale are listed in Table 3. The BPRS score was 54.69 ± 11.48 (anxiety depression factor score was 3.88 ± 0.47; the lacking active factor score was 3.11 ± 0.82; thinking disturbance factor score was 2.91 ± 1.23; activity factor score was 2.06 ± 0.66; the hostility factor score was 2.98 ± 1.17), self-knowledge impairment score was 2.88 ± 0.96, inability to work score was 4.25 ± 1.13, HAMD score was 30.00 ± 11.06, HAMA score was 18.06 ± 5.77, MMSE score was 28.06 ± 2.29 (two patients scored < 27), and MoCA score was 25.06 ± 3.40 (seven patients scored < 26).

**Table 3:** Neuropsychological rating scale.

**Rating scale**		**Score**
BPRS factors	Anxiety depression factor	3.88 ± 0.47
	The lacking active factor	3.11 ± 0.82
	The thinking disturbance factor	2.91 ± 1.23
	Activity factor	2.06 ± 0.66
	The hostility factor	2.98 ± 1.17
BPRS score		54.69 ± 11.48
HAMD		30.00 ± 11.06
HAMA		18.06 ± 5.77
MMSE		28.06 ± 2.29
MoCA		25.06 ± 3.40

### SPECT studies

**Figure 1:**
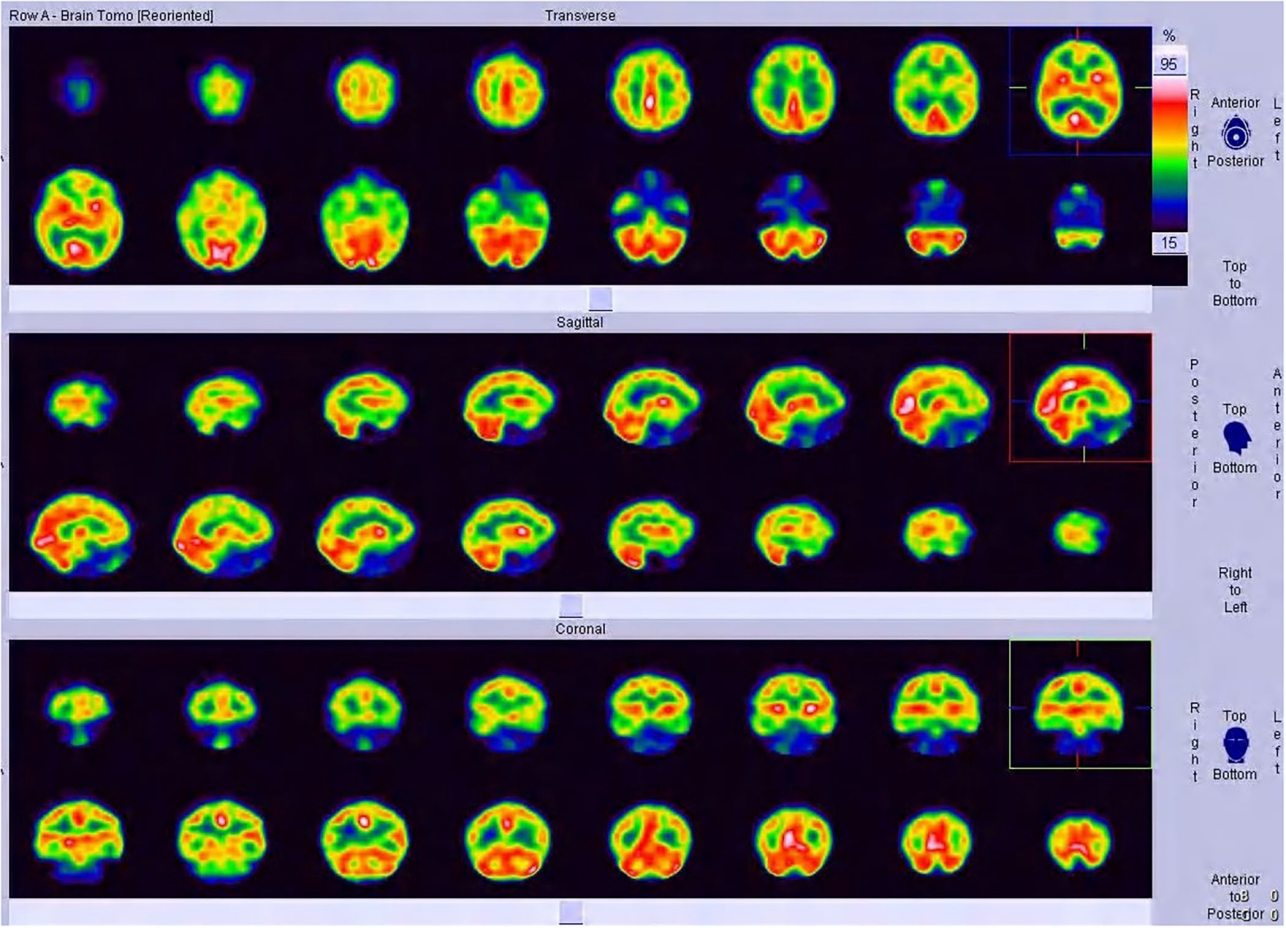
SPECT images of Case 2, a 19-year-old man.

**Figure 2:**
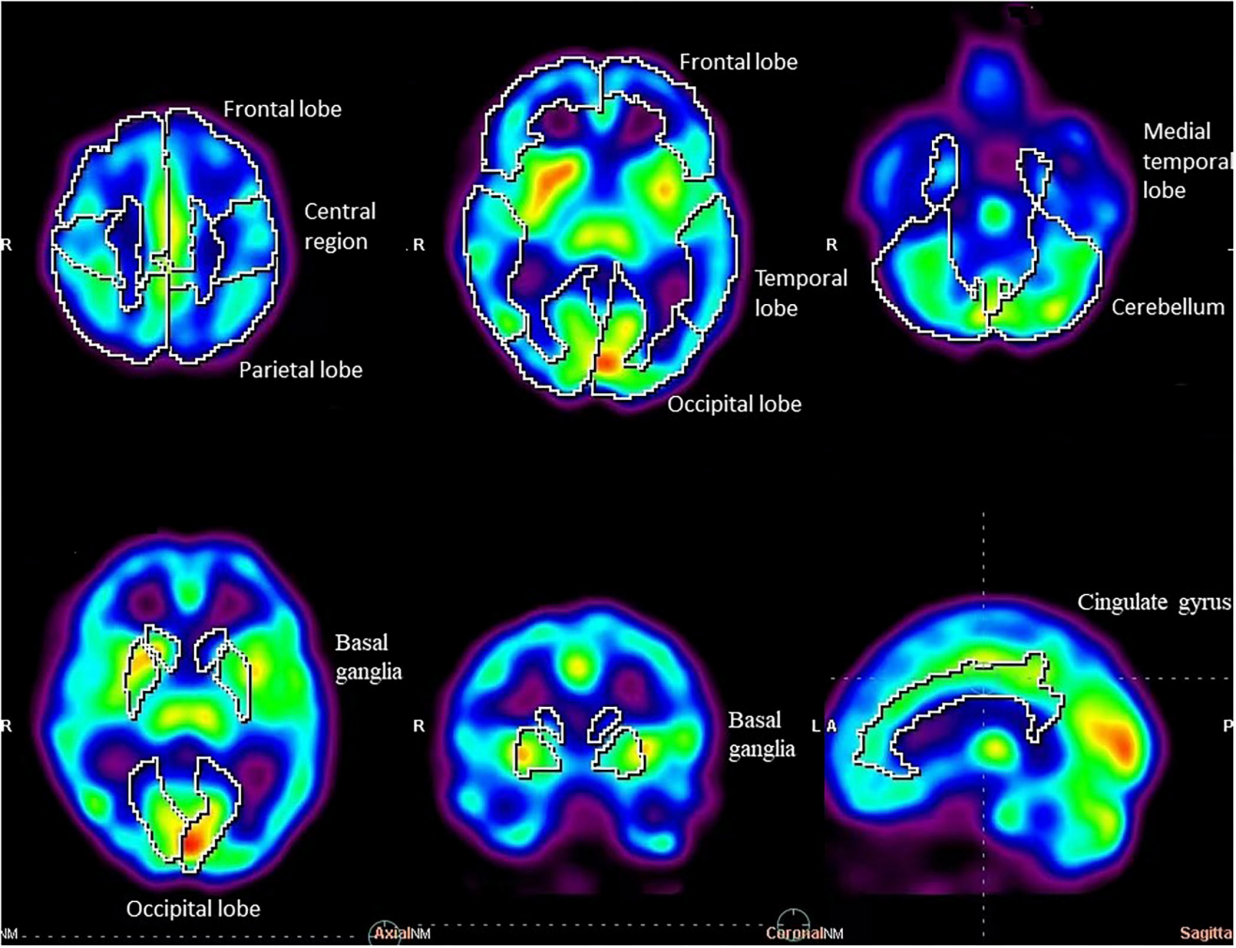
SPECT standard regions of case 15, a 23-year-old woman.

Regional uptake statistics of the ^99*m*^TC-ECD between patients and normal people of the same age-group from the background software database are given in Table 4. No statistical significance was found in the *t*-test results of left and right uptake statistics (*P* > 0.05). No significant difference was found between brain regions (*F* = 5.919, *P* < 0.01). Significant differences were identified in the changes in rCBF in different brain regions (*P* < 0.01). Although brain MRI was normal in all patients, SPECT showed hypoperfusion in the frontal lobe (9/16, 56.25%) and the temporal lobe (12/16, 75.00%), which is consistent with the clinical findings (Table 5, Figures 1, 2).

**Table 4:** Regional uptake statistics of ^99m^TC-ECD between patients and normal people of the same age group.

**ROI**	**Left (Mean ± SEM)**	**Right (Mean ± SEM)**	**Average (Mean ± SEM)**	**Pearson correlation**
Basal ganglia	−1.094, 2.519	−1.200, 2.338	−1.147, 2.391	0.942
Central region	0.350, 1.116	0.694, 0.990	0.522, 1.052	0.681
Cerebellum	−0.638, 1.480	−0.469, 1.940	−0.553, 1.699	0.907
Cingulate and paracingulate gyri	0.59375, 1.885	0.543, 2.202	0.568, 2.017	0.747
Frontal lobe	−1.575, 1.598	−1.925, 1.593	−1.743, 1.579	0.792
Medial temporal lobe	0.237, 1.862	−0.018, 2.660	0.109, 2.262	0.912
Occipital lobe	−0.793, 1.958	−0.775, 1.688	−0.784, 1.799	0.579
Parietal lobe	0.206, 2.063	0.400, 1.559	0.303, 1.801	0.800
Temporal lobe	−2.05, 1.580	−1.662, 0.879	−1.856, 1.273	0.692

**Table 5:** Regional cerebral perfusion changes of N_2_O abuse resulting in psychiatric disorders.

**ROI**	**Numbers (%)**		
	**No change**	**Increased perfusion**	**Decreased perfusion**
Basal ganglia	10 (62.50%)	1 (6.25%)	5 (31.25%)
Central region	12 (75.00%)	4 (25.00%)	0 (0.00%)
Cerebellum	9 (56.25%)	2 (12.50%)	5 (31.25%)
Cingulate and paracingulate gyri	6 (37.50%)	6 (37.50%)	4 (25.00%)
Frontal lobe	7 (43.75%)	0 (0.0%)	9 (56.25%)
Medial temporal lobe	9 (56.25%)	4 (25.00%)	3 (18.75%)
Occipital lobe	8 (50.00%)	3 (18.75%)	5 (31.25%)
Parietal lobe	9 (56.25%)	4 (25.00%)	3 (18.75%)
Temporal lobe	4 (25.00%)	0 (0.0%)	12 (75.00%)

## Discussion

N_2_O is colloquially known as “hippy crack” or “laughing gas.” It is increasingly taken for recreational purposes for its euphoric and relaxing effects and hallucinogenic properties. The abuse of N_2_O is a significant public health concern, which predominantly affects adolescents. Although the subacute combined degeneration of the spinal cord and peripheral neuropathy caused by N_2_O abuse are gradually concerned as the most common neurological damage (3, 4), psychiatric symptoms and cognitive dysfunction caused by N_2_O abuse have not received much attention (11, 12). All the patients enrolled in this study consumed large amounts of N_2_O for a long time before the onset of neuropsychiatric symptoms. Their psychiatric symptoms included emotional symptoms (e.g., mania, depression, anxiety, and fear), psychotic symptoms (hallucinations and delusions), personality changes, and impulsive and aggressive behavior, in line with previous reports (13). The anxiety depression factor score was the highest in the BPRS score, which was consistent with the HAMD and HAMA scores, suggesting that the patients must have symptoms of anxiety and depression. Thinking factors (e.g., disorder of concept, exaggeration, hallucinations, and abnormal thinking content) have high factor scores, indicating that patients with positive psychotic symptoms are prominent, and affect self-awareness and ability to work and study. The lack of vitality factor scores in the BPRS scores indicates that in addition to the positive symptoms, negative symptoms are also more prominent. This is not reported in previous studies. The relationship between negative symptoms and long-term prognosis and brain function needs further follow-up.

Of the 16 patients, 15 patients and their families complained of unresponsiveness and decreased memory, and seven of them had a MoCA score < 26, indicating that there was a decline in cognitive function (e.g., memory loss, inattention, and executive function decline). Both psychiatric symptoms and cognitive dysfunction result in the decline of work and life abilities.

Chronic N_2_O poisoning is related to the interference of vitamin B12, inactivates methionine synthase, interferes with myelin anabolic metabolism, and also results in the accumulation of homocysteine (14). High levels of homocysteine cause oxidative stress and mitochondrial dysfunction, leading to nerve demyelination (15). Homocysteine activates N-methyl-D-aspartate receptors on neuronal cell membranes and induces calcium ions to flow into neurons and mitochondrial calcium overload, eventually leading to neuronal necrosis and apoptosis (16). Of the 16 patients. 11 patients in this study had elevated serum homocysteine levels and only four of them had decreased levels of vitamin B12, which may be related to the nine patients who had self-medicated with methylcobalamin before their visit to our department. This result suggests that elevated serum levels of homocysteine is a more sensitive indicator than decreased serum levels of vitamin B12 for diagnosis (14).

Although the brain MRI of the patients enrolled in this study showed that the brain structure was normal, rCBF changes were different in different brain regions in this study. Local decreased or increased blood perfusion was observed in several brain regions, rather than diffuse decreased blood perfusion. The main findings were hypoperfusion in the frontal lobe and the temporal lobe, which is in accordance with the clinical manifestations of mental behavior abnormalities and cognitive functional decline caused by N_2_O abuse.

Similar to ketamine, N_2_O, as an NMDAR antagonist, can reduce the signaling of excitatory glutamate neurons, change the structure and function of hippocampal synapses, and cause learning and memory disorders. Short-term N_2_O exposure can cause reversible vacuolation of neurons, while long-term abuse can lead to neuron death (17). Psychiatric symptoms and cognitive decline in these patients may be dominated by this injury mechanism. Therefore, it is worth further studying the changes in neurotransmitters caused by N_2_O abuse and how they affect the learning and memory functions of adolescents.

In the treatment of patients with neuropsychiatric symptoms caused by N_2_O abuse, in addition to supplying vitamin B12 for nerve repair, we should also perform neuropsychological testing in time to strengthen interventions for mental symptoms and enhance patient compliance, which will not only improve the prognosis of patients but also help prevent relapse (18).

Previous studies have shown that N_2_O abuse may induce manic relapse in patients with mood disorders (19). The occurrence of psychiatric symptoms observed in this study is consistent with the clinical characteristics of substance-induced mental disorders, which indicate that N_2_O abuse can induce mental disorders. Future studies should expand the sample size to further explore the pathogenesis and disease characteristics of N_2_O-induced mental disorders.

As most of the patients reported initiation of N_2_O use in late adolescence or early adulthood, which involves risks to their still developing brain (20), education about N_2_O abuse is necessary to prevent impaired brain development. Follow-up and further investigation of the possible effects of N_2_O on brain development in young people will lead to meaningful prevention strategies.

The limitation of our study is the small sample size. We will continue to collect cases and expand the sample size to further explore the pathogenesis of N_2_O-induced neuropsychiatric damage.

## Conclusion

This was the first study to demonstrate the obvious effect of N_2_O abuse on CBF in patients with neuropsychiatric symptoms. CBF perfusion imaging is helpful in detecting the changes in rCBF in patients with N_2_O abuse, thus indicating changes in local brain functional activity in an early stage.

## Data availability statement

The original contributions presented in the study are included in the article/supplementary material, further inquiries can be directed to the corresponding author/s.

## Ethics statement

The study involving human participants was approved by the Medical Ethics Committee of China–Japan Friendship Hospital. The trial number is: 2018-34-K25. Written informed consent was obtained from all the participants or his/her parents/legal representatives (for participant under 18 years old).

## Author contributions

FL and CT: have full access to all the data in the study and take responsibility for the integrity of the data and the accuracy of the data analysis. LW, FL, and CT: study concept and design and study supervision. LW, QW, MD, and XD: acquisition of data. CT, RW, and ZL: neuropsychological rating. WH: magnetic resonance imaging. LY and YZ: SPECT/CT imaging. LW, QW, and FL: analysis and interpretation of data. LW and FL: drafting of the manuscript. LW: funding acquisition. All authors critical revision of the manuscript for important intellectual content. All authors read and approved the final manuscript.

## Funding

This study was supported by research grants from the First Hospital of Tsinghua, University Pilot Funds (LH-02).

## Conflict of interest

The authors declare that the research was conducted in the absence of any commercial or financial relationships that could be construed as a potential conflict of interest.

## Publisher's note

All claims expressed in this article are solely those of the authors and do not necessarily represent those of their affiliated organizations, or those of the publisher, the editors and the reviewers. Any product that may be evaluated in this article, or claim that may be made by its manufacturer, is not guaranteed or endorsed by the publisher.
